# Airborne Hyperspectral Imagery for Band Selection Using Moth–Flame Metaheuristic Optimization

**DOI:** 10.3390/jimaging8050126

**Published:** 2022-04-26

**Authors:** Raju Anand, Sathishkumar Samiaappan, Shanmugham Veni, Ethan Worch, Meilun Zhou

**Affiliations:** 1 Department of Electronics and Communication Engineering, Amrita School of Engineering, Amrita Vishwa Vidyapeetham, Coimbatore 641112, India; 2 Geosystems Research Institute, Mississippi State University, Starkville, MS 39759, USA; sathish@gri.msstate.edu (S.S.); eworch99@gmail.com (E.W.); zhou.m@ufl.edu (M.Z.)

**Keywords:** moth–flame, optimization, hyperspectral image, classifier, particle swarm, genetic algorithm, cuckoo search

## Abstract

In this research, we study a new metaheuristic algorithm called Moth–Flame Optimization (MFO) for hyperspectral band selection. With the hundreds of highly correlated narrow spectral bands, the number of training samples required to train a statistical classifier is high. Thus, the problem is to select a subset of bands without compromising the classification accuracy. One of the ways to solve this problem is to model an objective function that measures class separability and utilize it to arrive at a subset of bands. In this research, we studied MFO to select optimal spectral bands for classification. MFO is inspired by the behavior of moths with respect to flames, which is the navigation method of moths in nature called transverse orientation. In MFO, a moth navigates the search space through a process called transverse orientation by keeping a constant angle with the Moon, which is a compelling strategy for traveling long distances in a straight line, considering that the Moon’s distance from the moth is considerably long. Our research tested MFO on three benchmark hyperspectral datasets—Indian Pines, University of Pavia, and Salinas. MFO produced an Overall Accuracy (OA) of 88.98%, 94.85%, and 97.17%, respectively, on the three datasets. Our experimental results indicate that MFO produces better OA and Kappa when compared to state-of-the-art band selection algorithms such as particle swarm optimization, grey wolf, cuckoo search, and genetic algorithms. The analysis results prove that the proposed approach effectively addresses the spectral band selection problem and provides a high classification accuracy.

## 1. Introduction

Hyperspectral Imaging (HSI) sensors can acquire detailed reflectance information from narrow spectral bands in the visible, Near-Infrared (NIR), mid-IR, and thermal IR portions of the light spectrum [1]. HSI sensors can collect hundreds of bands with a high spectral resolution, having near-continuous spectral reflectance information for every pixel. This enables researchers to characterize different ground materials which is otherwise impossible with optical or multispectral remote sensing. The primary limitation of HSI is its higher dimensionality and the resulting Hughes phenomenon [2]. To alleviate the Hughes phenomenon and reduce the computation time of hyperspectral analysis, the dimensionality of the data needs to be reduced without losing information. Dimensionality reduction can be achieved in two ways: (1) Feature extraction: this needs a transform to convert a high-dimensional HSI cube into a lower-dimensional space where the originaldata information is lost [3,4]; (2) feature selection or band selection: this reduces the dimensionality to optimize the classification accuracy with a limited sample size. Feature selection selects the ideal mix of bands for supervised learning, utilizing the underlying optimization techniques [5]. Approximation algorithms that are used to find subsets are useful sub-optimal solutions, since finding optimal solutions through exhaustive searches requires increased processing time and computational complexity. Due to this, band selection is the perfect mechanism to reduce the dimensionality and maintain the statistical confidence of the Hughes phenomenon [6].

The balance between exploration and exploitation is a critical aspect in attaining global optimization for band selection. The goal is to cover the entire search space to avoid missing the genuine optima; nevertheless, to discover the genuine optima, the search should zero in on a specific good part of the solution space. A variety of “no free lunch” (NFL) theorems have been presented, establishing that any improved performance over one class of tasks is countered by a reduced performance over another [7]. This theorems lead to a geometric view of what it means for an algorithm to be well performing and appropriate for an optimization problem [8]. The no free lunch theorem demonstrates that an optimization strategy that works well for one type of issue may not work well for another. This theorem states that some may perform better for one sort of data when compared to other metaheuristic techniques. As a result, it is worthwhile to investigate each suggested metaheuristic strategy for HSI band selection. Many nature-inspired metaheuristics, such as Particle Swarm Optimization (PSO), Genetic Algorithm Optimization (GAO), Cuckoo Search Optimization (CSO), differential evolution, and so on, have already been well studied for HSI band selection.

The contributions of this research are as follows:

1. We propose an MFO-based algorithm for hyperspectral band selection.
2. We implemented and tested MFO-based hyperspectral band selection for three benchmark datasets.
3. We compared the performance of MFO with three state-of-the-art metaheuristic band selection methods.

PSO mimics the navigation and foraging of a flock of birds or a school of fish [9]. PSO analyzes the HSI data models for the optimization of band selection. The approach is built on an abstraction of the selection process and was introduced by [10,11]. PSO has difficulty with respect to designing parameters and problems due to the scattering of the data points in a three-dimensional space. It also becomes stuck in local minima, in particular with complex optimum solutions. To overcome this, the genetic algorithm enables the global optimum to be large. In contrast, conventional approaches for optimization will converge towards the local minimum without the global optimum. Genetic algorithms are used to find the optimal set of parameters with less cost. GAO requires little knowledge about the optimization problem, but the creation of an objective function of optimization application is complex and requires more computational resources (i.e., it is time-consuming). CSO was inspired by the obligate brood parasitism of some cuckoo species by laying their eggs in the nests of other host birds (of other species) [12,13]. Some host birds can engage in direct conflict with the intruding cuckoos (i.e., if a host bird discovers the eggs are not its own, it will either throw these foreign eggs out or simply abandon its nest and build a new nest elsewhere). There are three major operators is to find the best optimum values of the bands. The first process is the Levy flight; it generates a new solution (eggs) by perturbing the current fitness value [14]. The second operator is the host bird, which can throw the eggs away/abandon the nest (with a fraction $p_{a}=\left[0,1\right]$ and build an entirely new nest. The third operator makes a selection of the optimum bands, which are optimized with the help of the second operator. The CS approach is not ideal because it quickly falls into the optimal local solutions and has a slow convergence rate [15]. We need a global optimization technique such as MFO to overcome these issues. MFO benefits from being simple to understand and apply, and it provides a high convergence rate [16]. Nonetheless, the literature indicates that the MFO algorithm has the potential for development. As a result, several researchers have attempted to develop the algorithm in various ways during the last two years.

Metaheuristic algorithms have been used as promising methods for solving many problems over the last decade. Many metaheuristic algorithms, however, may provide unsatisfactory performance due to slow or premature convergence. As a result, determining how to develop algorithms that balance exploration and exploitation while precisely locating the appropriate hyperspectral band remains a challenge. To solve hyperspectral band selection problems, this paper proposes a new global optimization algorithm based on MFO. The main source of inspiration for this algorithm is the transverse orientation navigation method used by moths in nature. Moths fly at night by maintaining a fixed angle with respect to the Moon, which is a very effective mechanism for traveling long distances in a straight line. These beautiful insects, however, are trapped in a spiral path around artificial lights. We attempted to address the phenomenon of the MFO algorithm’s slow convergence and low precision. MFO is used to maintain a high level of global exploration and an effective balance between global and local searches. Although evolutionary multi-objective optimization approaches have recently been proposed to simultaneously optimize the criteria, they are unable to manage the global exploration versus local exploitation capabilities in the search space for the hyperspectral feature selection problem. Thus, a unique discrete sine-cosine-algorithm-based multi-objective feature selection strategy for hyperspectral imaging is proposed in this work. The suggested method creates a novel and effective framework for multi-objective hyperspectral feature selection. The framework models the ratio of the Jeffries–Matusita distance to mutual information to minimize redundancy and maximize the relevance of the selected feature subset. In addition, the variance of band selection is used to maximize the amount of information.

Evolutionary calculation is a natural evolution-inspired computational intelligence approach. The evolutionary calculation method begins by generating a random population of individuals that representto get a optimization solutions. The first population could be generated at random or by feeding it into the algorithm. Individuals are evaluated using a fitness function, and the output of the function indicates how well the individual solves or approaches solving the problem. Then, several natural evolution-inspired operators, such as crossover, mutation, selection, and reproduction, are applied to individuals. A new population is created based on the fitness values of newly evolved individuals. Some individuals are eliminated because the population size must be maintained in the same way as it is in nature. This method is repeated until the termination requirement is satisfied. The most commonly used condition for stopping the algorithm is reaching the number of defined generations. As the answer, the best individual with the highest fitness value is chosen. Every search algorithm must address search space exploration and exploitation. Exploration is the process of visiting completely new regions of a search space, whereas exploitation is the process of visiting areas of a search space that are close to previously visited places. A search algorithm must achieve a suitable balance between exploration and exploitation in order to be successful. In supervised machine learning, algorithms have achieved good performance based on the number of examples in the training set, the dimensions of the feature space, the correlated features, and overcoming overfitting problems. These are just a few factors on which the selection of the algorithm may depend. Based on this, we used three different supervised machine learning algorithms, Random Forest (RF), K-Nearest Neighbor (KNN), and Support Vector Machine (SVM). The derived spectral and spatial information for an effective classification was then learned using RF, KNN, and SVM. The problem with local optima was solved with the MFO algorithm. This optimization approach has been applied efficiently in various fields to find an optimum solution.

## 2. Materials and Methods

### Moth–Flame Optimization Algorithm

MFO navigates through a process known as transverse orientation. A moth flies in this manner by keeping a constant angle with the Moon, which is a compelling strategy for traveling long distances in a straight line, considering the Moon’s distance from the moth, which is considerably long [17]. Artificial lights fool moths, causing them to exhibit certain habits. The proximity of such illumination to the moth and maintaining a relative angle to the light source result in a spiral flying direction of the moths. Moths travel in a logarithmic spiral through flames in the MFO algorithm, then converge on the flame. Moths were assumed to be possible solution candidates in our MFO technique, and the spatial coordinates were re-used as the input variables for the flame problem. As a result, the moth’s position vectors allowed them to fly in the 1D, 2D, 3D, or hyper-dimensional space. Because the MFO algorithm uses a population-based approach, the collection of moths is represented in the following matrix.

(1) $$M=\left[\begin{array}{ccc} m_{1,1}\;\;\;m_{1,2} & \cdots & m_{1,d}\\ \vdots & \ddots & \vdots \\ m_{n,1}\;\;{\;m}_{n,2} & \cdots & m_{n,d} \end{array}\right]$$

The return value of the objective function for each moth is the fitness value. When the fitness function receives a position vector (such as the first row of the matrix M), it assigns the fitness function output to the appropriate moth (OM). Flames are an important part of MFO. The following is an example of a flames matrix:

(2) $$F=\left[\begin{array}{ccc} F_{1,1}\;\;\;F_{1,2} & \cdots & F_{1,d}\\ \vdots & \ddots & \vdots \\ F_{n,1}\;\;{\;F}_{n,2} & \cdots & F_{n,d} \end{array}\right]$$

where *n* is the number of moths and d is the number of variables (dimensions). For all the moths, we assumed that there was an array for sorting the corresponding fitness function as follows.

(3) $$OM=\left[\begin{array}{c} {\mathrm{OM}}_{1}\\ {\mathrm{OM}}_{2}\\ .\\ .\\ {\mathrm{OM}}_{n} \end{array}\right],OF=\left[\begin{array}{c} {\mathrm{OF}}_{1}\\ {\mathrm{OF}}_{2}\\ .\\ .\\ {\mathrm{OF}}_{n} \end{array}\right]$$

**Figure 1:**
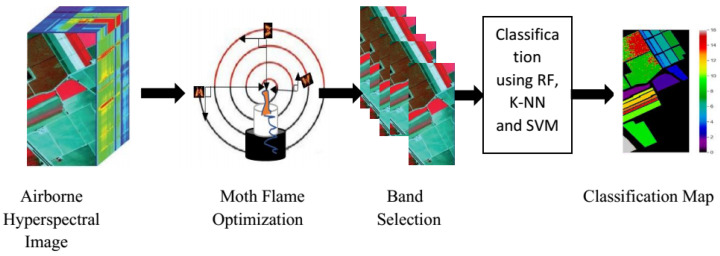
Schematic interpretation of the proposed method.

**Figure 2:**
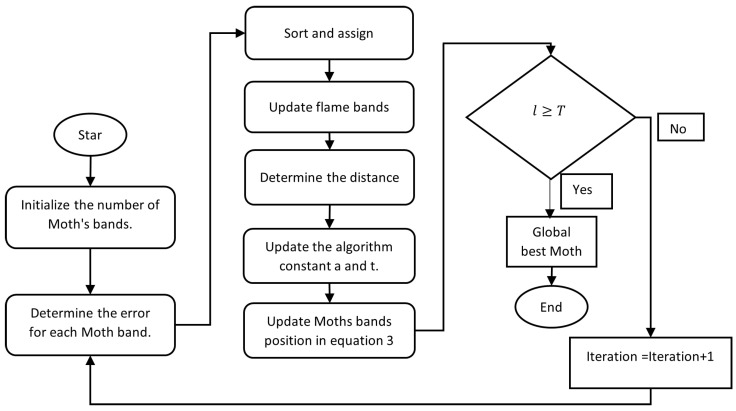
Proposed band selection system with the MFO algorithm.

In MFO, local and global searches for selecting bands from the hyperspectral band search space help produce quality classification maps. Machine learning algorithms are needed to classify hyperspectral data based on features. The proposed method for the optimized band selection method is depicted in Figure 1. The random parameter (T) accelerates convergence during generation, which varies from −1 to 1 [16,17]. The flow chart of the MFO algorithm is shown in Figure 2. When dealing with non-linear objective functions, the original PSO, CSO, and GA all suffer from the problem of local optima. Aside from that, because of the randomness, the convergence speed and precision are both very low. We suggested the MFO technique in order to solve all of these disadvantages, while also increasing the efficiency of the solution, as well as the pace of convergence [18]. The original MFO, on the other hand, makes use of the ability to travel in a spiral pattern in order to achieve exploitation rather than simply exploring the solution space.

The first approach, spiral behavior, can be understood as the moths trying to catch the flame considering it as prey through a spiral path in a transverse orientation [16]. The corresponding model is shown in Equation (5). To be more accurate, the MFO bands explore all available classification maps. According to Equation (5), the MFO bands encircle the maps or form a spiral in a transverse direction depending on the distance between the actual position of the band and the best positions obtained so far. The optimal band selection using the MFO is given in Algorithm 1.

**Algorithm 1: Optimal band selection from airborne HSI using moth–flame optimization.**

**Input**: Train and test datasets from $H_{cube}$

**Output**: Band collection derived from the HSI data cube using MFO’s global optimum location.
Procedure MFO Algorithm
  Moths are generated randomly at first to populate the feasible search space
  Evaluate and classify the fitness of the entire population
  Equal to the sorted population in flames
  While iteration < max iteration  The following equation can be used to calculate the flame number.

(4)
$$FlameNumber=round\left(N-l*\frac{N-1}{T}\right)$$

where *l* is the current iteration, *N* is the maximum flame number, and *T* is the maximum number of iterations.
The distance $D_{i}$ between the ith moth $M_{i}$ concerning its corresponding jth flame $F_{j}$ can be obtained from:
(5)
$$D_{i}=\;\left|\;F_{j}-M_{j}\right|$$

Update the value of constant a and t.

(6)
$$a=-1\;+\;l*\frac{-1}{T}$$


(7)
$$t=\left(a-1\right)*rand\;+\;1\;$$

where *t* is a random number between [−1, 1]. Maintain the direction of the moth and its associated flame $\left(F_{j}\right)$ . We used the spiral function (*S*), which simulates the moth’s transverse orientation around the Moon using the following equation.

(8)
$$S\left(M_{i},F_{j}\right)=D_{i}*e^{\left\{bt\right\}}*\cos\left(2*pi*t\right)+\;F_{j}$$

where *b* is the spiral shape constant.
Update and sort the fitness for all search agents.
Update the flames.
Iteration = Iteration + 1.
End while
  To find the global best position


The moths are modified using the output of Algorithm 1, which generates a collection of hyperspectral bands via MFO. Then, after selecting representative bands, the initial hyperspectral cube $H\in R^{w*h*\lambda }$ , where w and h are the spatial information of the HSI and λ denotes the spectral information, and it becomes $H_{MFO}$ ∈ $R^{w*h*\lambda }$ where r denotes the bands obtained from MFO and r<λ . The main source of inspiration for this algorithm is the transverse orientation navigation method used by moths in nature. Moths fly at night by maintaining a fixed angle with respect to the Moon, which is a very effective mechanism for traveling long distances in a straight line. These beautiful insects, however, are trapped in a spiral path around artificial lights. Here, the moth has coordinated values indicating the solution of the optimization problem or the group of band combinations. The moth population has a predetermined number of moth positions. This flame will act as the objective function or fitness function for optimization band selection. The moth position is update based on the number of bands and each iterations. The proposed paradigm is made up of two opposing objective functions. One assesses the amount of information, while the other measures the level of redundancy in the selected bands. The two elements are quantified by this model, allowing them to be optimized concurrently. A new multi-objective immune method is built to accommodate the features of hyperspectral data in order to optimize this model [19].

## 3. Classification Methods

Random forest is a classification algorithm that uses many decision tree models built on different sets of bootstrapped features [20]. This algorithm works with the following steps: Bootstrap the training set multiple times. The algorithm adopts a new set to build a single tree in the ensemble during each bootstrap. Whenever the tree sample is split, a portion of features is selected randomly in the training sets to find the best split variable, and new features are evaluated. The KNN algorithm is a non-parametric lazy learning algorithm [21]. Its purpose is to use a database in which the data points are separated into several classes to predict a new sample point classification. Support vector machines are now regarded as actual examples of “kernel Methods,” one of the critical areas in machine learning. SVM tries to map an input space into an output space using a nonlinear mapping function ϕ such that the problem of the data points becomes linearly separable in the output space [22]. When these points become linearly separable, then SVM discovers the optimal separating hyperplane.

### Dataset Description

**Figure 3:**
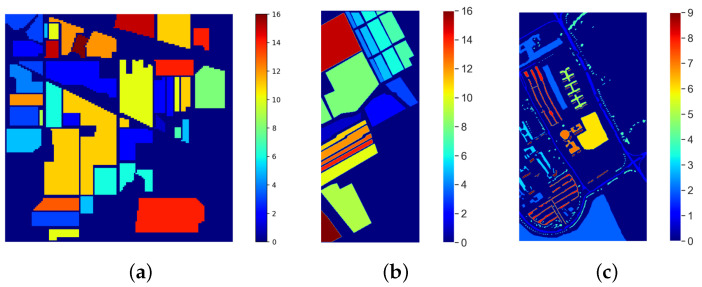
Ground truth map of three different benchmark airborne hyperspectral remote sensing scenes. (**a**) Indian Pines Ground Truth Hyperspectral Data, (**b**) Salinas Ground Truth Hyperspectral Data, (**c**) Pavia University Scene Ground Truth Hyperspectral Data.

The airborne hyperspectral datasets used in this experiment were the Indian Pines [23], Salinas [24], and University of Pavia images [25]. The ground truth of a sample image from these datasets is shown in Figure 3. The Indian Pines dataset was collected by an Airborne Visible/Infrared Imaging Spectrometer (AVIRIS) sensor. This dataset was taken in the northwest part of Indiana in the year 1992. Each sample has 224 spectral bands with a spatial dimension of 145 × 145 and ground truth data, as shown in Figure 3a. The Salinas dataset was taken in Salinas Valley, California, in the year 1998 using the AVIRIS sensor. These data have 224 spectral bands with a 512 × 217 spatial dimension with ground truth data and 17 different land cover classes, as shown in Figure 3b. The University of Pavia Scene dataset was collected using the Reflective Optics System Imaging Spectrometer (ROSIS) sensor. This dataset was taken in a flight campaign over Pavia, Northern Italy. These data have 103 spectral bands with a 610 × 340 spatial dimension with ground truth data and have 9 different classes, as shown in Figure 3c [26]. MFO can optimize images with multiple bands by selecting very few bands, and it is compared with other state-of-the-art methods in Table 1.

## 4. Experimental Results

The research work mainly focused on finding optimal bands in hyperspectral data [27], improving test samples’ classification accuracy and prediction rate.

The performance of the MFO algorithm was evaluated by using 20% of the samples for training and the remaining 80% of the samples for testing. The details of the training and testing samples for the three datasets are provided in Table 2.

### Classification Maps

**Figure 4:**
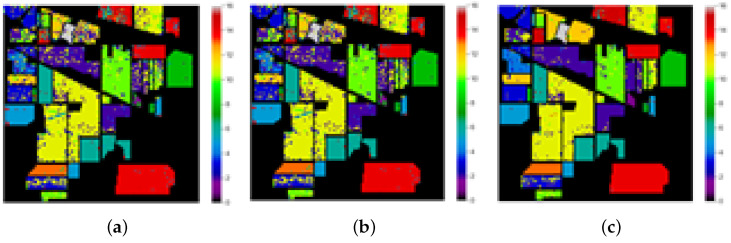

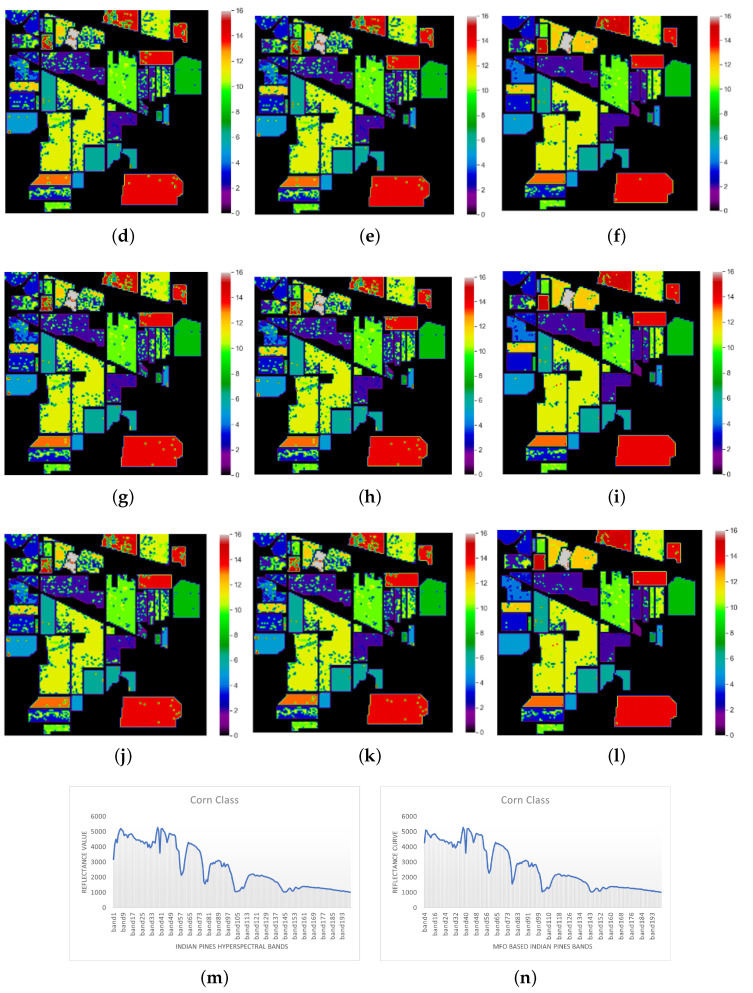
Classification maps of Indian Pines hyperspectral data: (**a**) PSO-RF, (**b**), PSO-KNN (**c**), PSO-SVM (**d**) GAO-RF, (**e**) GAO-KNN, (**f**) GAO-SVM, (**g**) CSO-RF, (**h**) CSO-KNN, (**i**) CSO-SVM, (**j**) MFO-RF, (**k**) MFO-KNN, (**l**) MFO-SVM, (**m**) spectral reflectance for original bands for corn class, and (**n**) spectral reflectance for MFO-based selected bands for corn class.

**Figure 5:**
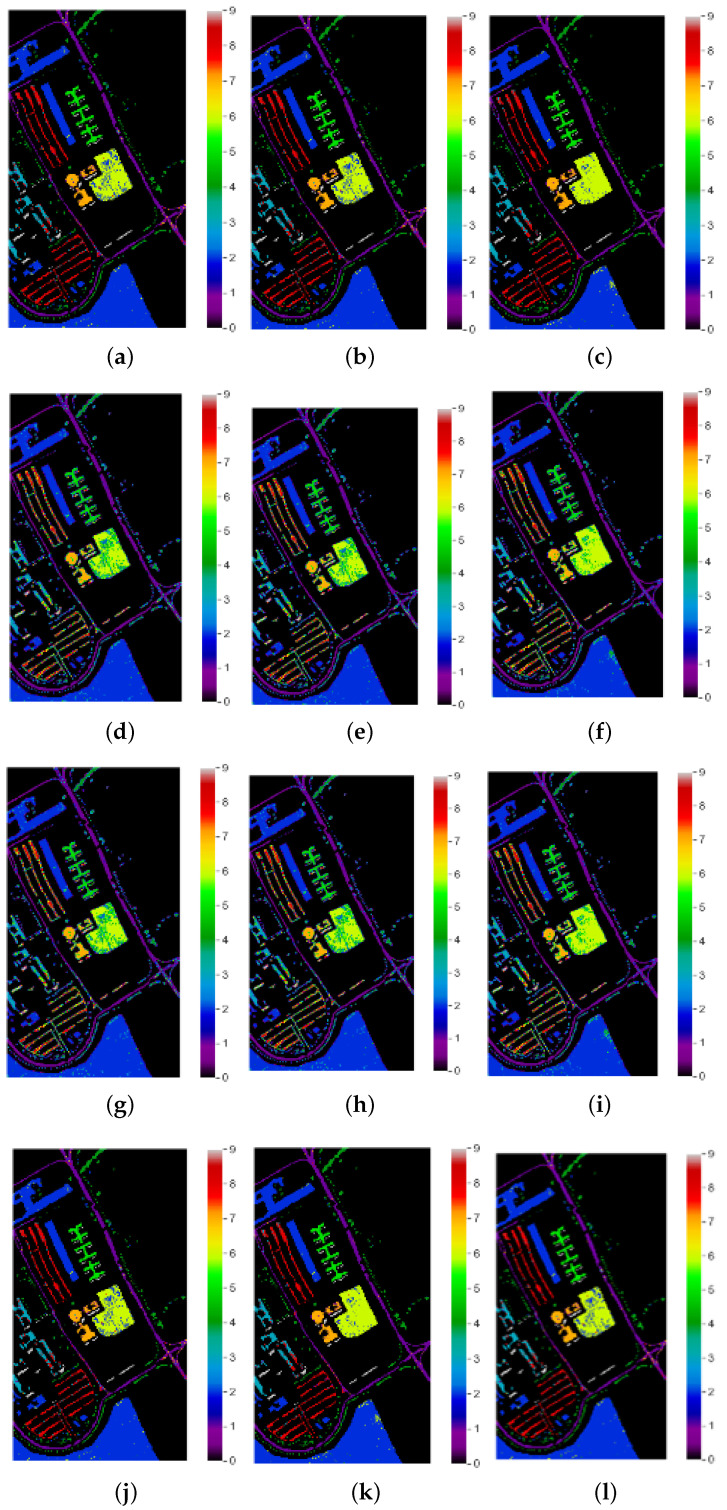
Classification maps of University of Pavia hyperspectral data: (**a**) PSO-RF, (**b**) PSO-KNN, (**c**) PSO-SVM, (**d**) GAO-RF, (**e**) GAO-KNN, (**f**) GAO-SVM, (**g**) CSO-RF, (**h**) CSO-KNN, (**i**) CSO-SVM, (**j**) MFO-RF, (**k**) MFO-KNN, and (**l**) MFO-SVM.

**Figure 6:**
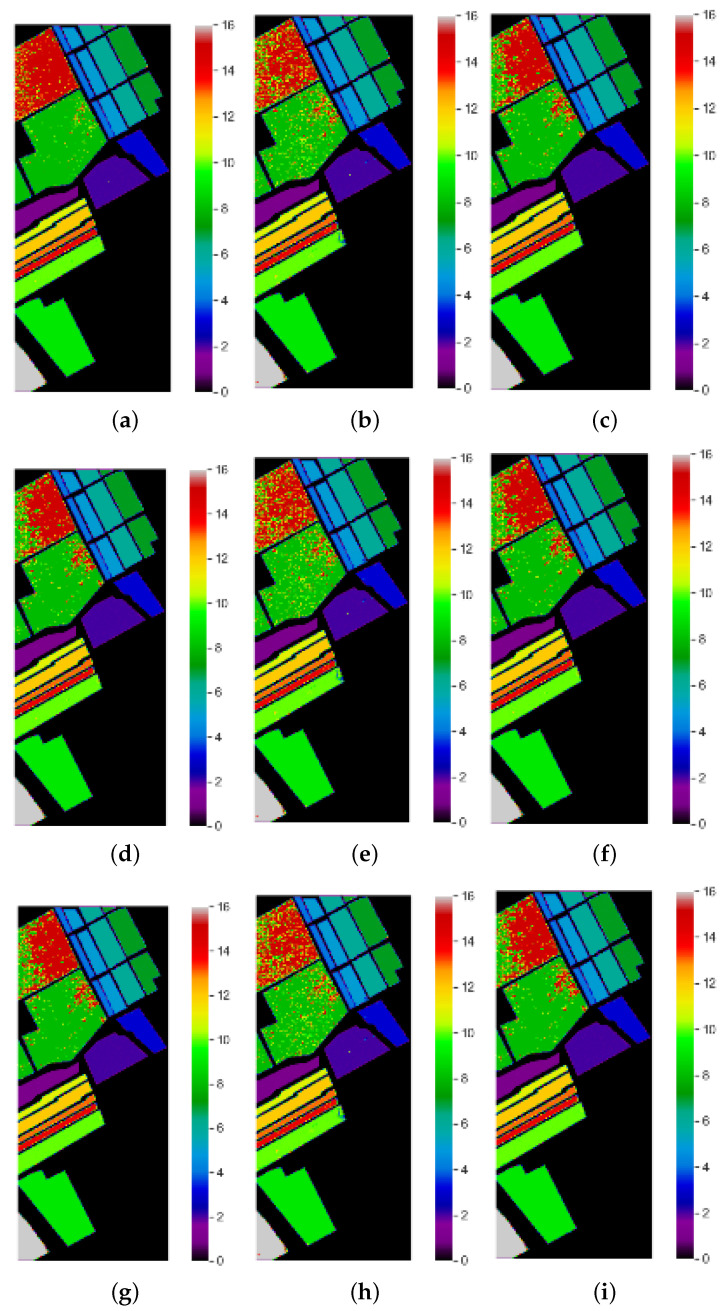

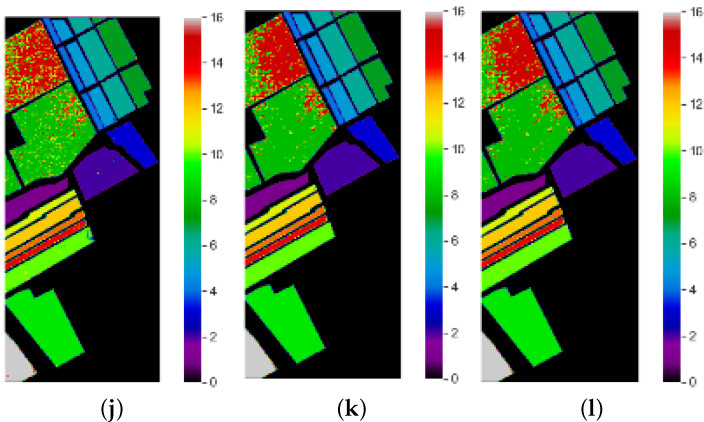
Classification maps of Salinas hyperspectral data:, (**a**) PSO-RF, (**b**) PSO-KNN, (**c**) PSO-SVM, (**d**) GAO-RF, (**e**) GAO-KNN, (**f**) GAO-SVM, (**g**) CSO-RF, (**h**) CSO-KNN, (**i**) CSO-SVM, (**j**) MFO-RF, (**k**) MFO-KNN, and (**l**) MFO-SVM.

This research proposed a method for band selection using MFO. The designation accuracy was used as the fitness function in this algorithm, and global bands were divided into sub-optimal bands based on the fitness values. The following subsection discusses different optimization techniques. PSO-based hyperspectral band selection was implemented, and it was tested with benchmarks of hyperspectral data. Good overall accuracy was achieved, but PSO has disadvantages, such as easily falling into local optima [28]. The band selection of HSI using the cuckoo search algorithm was implemented and tested with benchmark hyperspectral datasets. Here, the cuckoo search algorithm achieved good accuracy; however, it has two objective functions that need to be implemented, and it had slow convergence for multi-dimensional data [29]. Genetic algorithms (GAs) adopt probabilistic methods of search to minimize a given fitness or cost function. The main features of this optimization technology are that: (i) the algorithm does not deal with the parameters, but instead codes them; (ii) the optimal search works with a population of solution points; (iii) the derivatives of the fitness function are unknown; (iv) the algorithm uses laws of probabilistic transformation rather than deterministic ones for hyperspectral band selection [30]. The classification maps of the proposed approach and other state-of-the-art approaches for sample images from the Indian Pines, University of Pavia and Salinas datasets are depicted in Figure 4, Figure 5 and Figure 6, respectively. From Figure 4j–l, The proposed MFO band selection method is observed to classify the class grasstrees accurately using the RF, KNN, and SVM classifiers (Anand R, 2017) for the Indian Pines HSI. In the case of the KNN classifier, it is observed that the classes grasstrees, grass-pasture-mowed, oats, and hay-windrowed were classified accurately. The rate of misclassification in other classes was low, owing to the intelligent behavior of MFO. However, compared to these methods, SVM played a significant role in this research, especially MFO, because it provided a perfect classification for several classes such as alfalfa, class grasstrees, grass-pasture-mowed, hay-windrowed, oats, and wheat. From Figure 5j–l, the proposed moth–flame optimized band selection method is observed to classify the bare class soil accurately using the RF, KNN, and SVM classifiers for the University of Pavia hyperspectral data. Due to the mixed pixel problem in the hyperspectral cube, a portion of the self-blocking bricks was incorrectly classified as asphalt. Nevertheless, compared to the RF and KNN classifier methods, SVM played a major role in this research, especially MFO, because it provided good classification accuracy for the three classes bare soil, gravel, and trees. From Figure 6j–l, the proposed moth–flame optimized band selection method is observed to classify the class celery accurately using RF classifiers for the Salinas scene hyperspectral data. The classes of celery and lettuce romaine 7 wk were classified accurately using the KNN classifier. However, compared to the RF and KNN classifiers, SVM played a significant role in this research, especially MFO, because it classified many classes accurately such as broccoli green weeds 2, celery, corn senesced green weeds, and lettuce romaine 7 wk. This is because the selected bands were very useful to classify accurately. It was observed that samples of fallow rough plow, lettuce romaine 6 wk, and soil vineyard develop were misclassified because of the highly correlated pixel values.

## 5. Comparative Analysis of State-of-the-Art Approaches

The efficiency of the proposed method was compared with PSO [31], GA [32], and CSO [33]. Table 3 summarizes the suggested band selection method’s classwise accuracies over Indian Pines. The highest accuracy is denoted in bold. Alfalfa, grasstrees, grass-pasture-mowed, hay-windrowed, oats, and wheat were classified with 100% accuracy. This was accomplished by the intelligent behavior of the MFO’s band selection algorithm. The corn, grass-pasture, soybean-clean, and buildings grasstrees classes obtained accuracy values of 82.22%, 96.85%, 88.16%, and 78.21%, respectively. The accuracy of the other approaches was between 30% and 70%. The accuracy was achieved by extracting only optimized bands through MFO. Additionally, the remaining six classes exhibited high classification accuracy, while the remaining classes exhibited comparable performance to the other methods.

Table 4 summarizes the suggested band selection method’s classwise accuracy on the Pavia dataset. The highest accuracy is denoted in bold. Among the nine groups, MFO-based band selection outperformed all other approaches. Two of the classes, bare soil and trees, achieved a classification accuracy of 100%. Asphalt, meadows, and shadows all had high accuracies of 96.69%, 98.67%, and 93.21%, respectively, while the accuracy of the other approaches was in the range of 71.19 to 92.41%. Painted metal sheets were graded similarly by the PSO of RF and the MFO of KNN since they had a different spectral reflectance property compared to other types of reflectance.

Table 5 summarizes the suggested band selection method’s classwise accuracies over the Salinas dataset. The highest accuracy is denoted in bold. From the sixteen classes, four classes achieved a classification accuracy of 100.00% for the MFO-based bands. The accuracies of the untrained classes such as stubble, celery, and corn senesced green weeds were 99.94%, 99.72%, and 98.63%, respectively, and comparatively outperformed the other approaches. Three classes demonstrated competitive success using alternative approaches.

### 5.1. Comparative Analysis of State-of-the-Art Techniques with Respect to Overall and Average Accuracies

**Figure 7:**
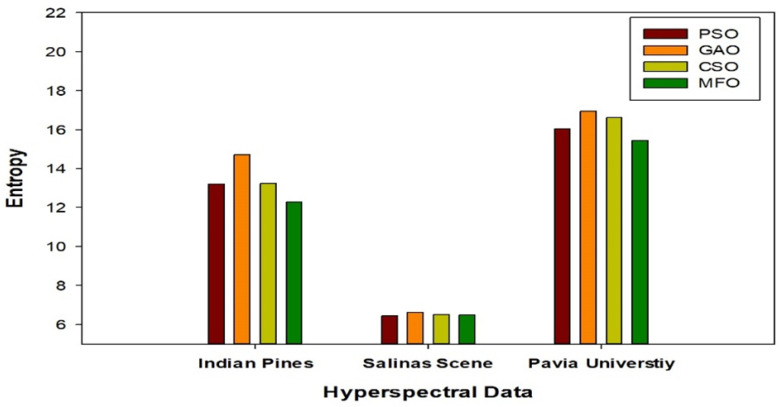
Quantitative analysis of selected bands’ average entropy for the three datasets with different optimization techniques.

**Figure 8:**
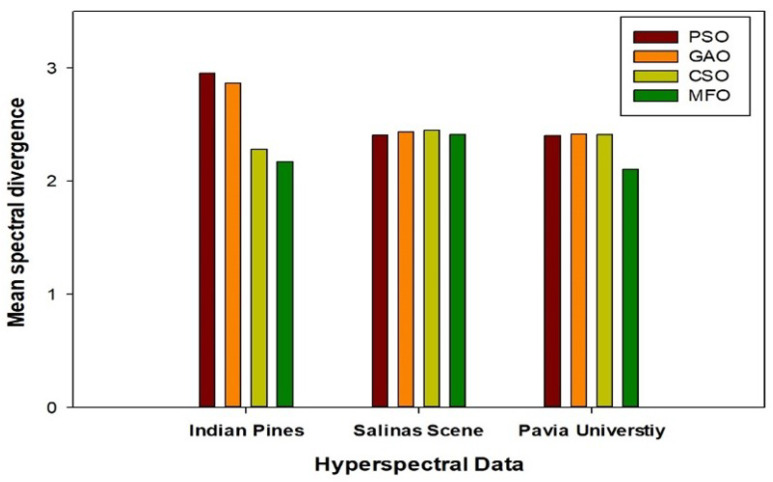
Quantitative analysis of selected bands’ mean spectral divergence for the three datasets with different optimization techniques.

**Figure 9:**
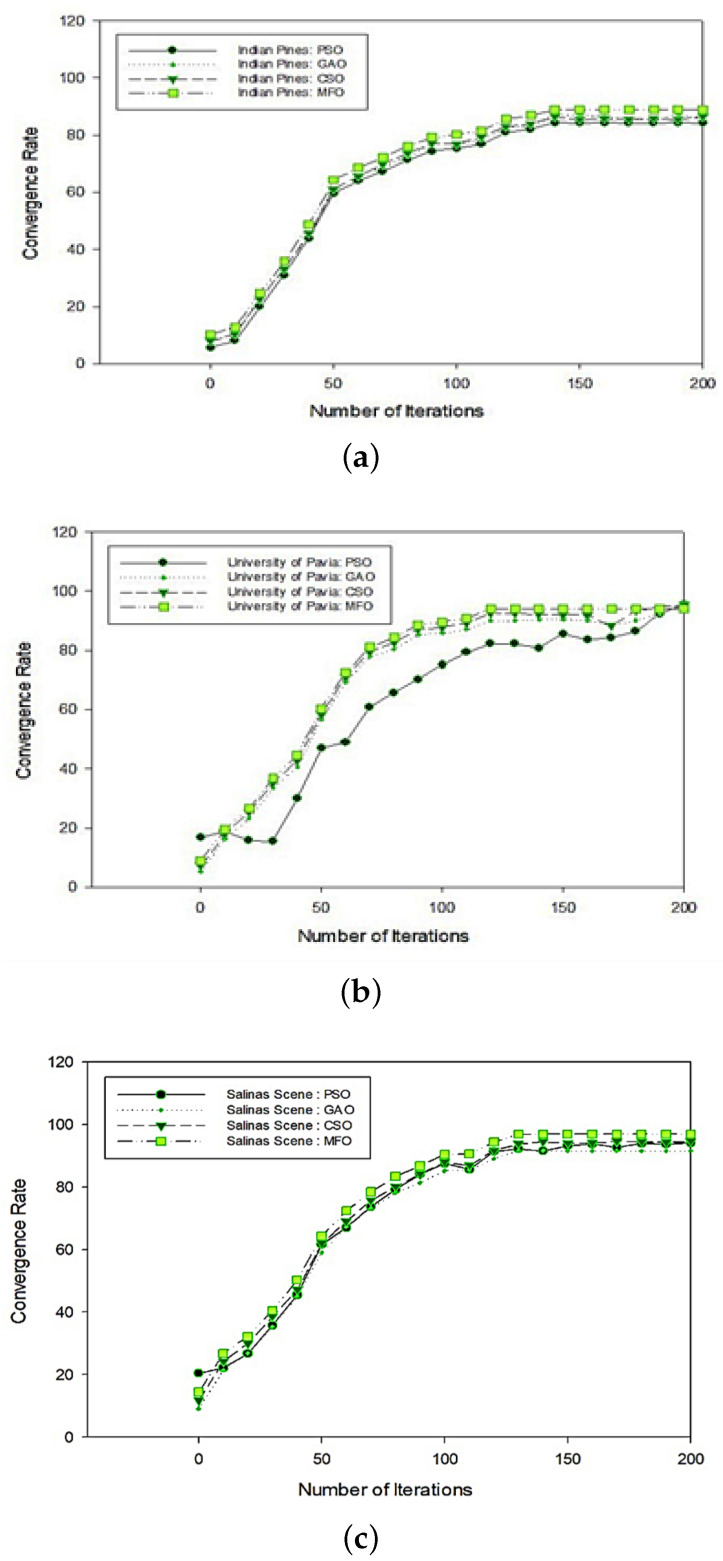
Proposed MFO convergence curve analysis over the number of iterations: (**a**) Indian Pines, (**b**) University of Pavia, and (**c**) Salinas.

On all datasets, the proposed MFO algorithm was efficient in attaining actual class marks. As shown in Table 6, the proposed method’s overall accuracy on the Indian Pines dataset was 88.98% for the SVM classifier. It varied between 75.00% and 88.90% with the other strategies. The average accuracy was 91.37 %, while it varied between 72.46% and 90.11% for the other approaches. Similarly, the average accuracy of the University of Pavia dataset was 96.94%, compared to 91.84 to 95.48% for other approaches. Additionally, the overall accuracy was 94.85%, compared to 89.95% to 93.53% for the other systems. Overall, the Salinas dataset collection accuracy was in the range of 91.99% to 95.45% with the average accuracy of 93.9%. The overall average accuracy was 97.17%, while the accuracy of the other approaches varied between 95.86 and 97.71%, as shown in Table 7 and Table 8, respectively. To validate each method, MFO had 30 search members, and 200 iterations were used. It should be emphasized that the number of moths should be selected empirically (or other candidates for solutions in other algorithms). The more fake moths are used, the more likely the global optimum is determined. However, 30 moths for addressing optimization issues is a reasonable number, and it can be decreased to 20 or 10 for costly situations. The entropy of the selected bands of the Indian Pines, Salinas Scene and University of Pavia hyperspectral data and the selected bands from different optimization techniques is shown in Figure 7. It can be seen from Figure 8 that the proposed moth–flame optimization techniques had an average entropy of 12.28157. The proposed moth–flame-based band section showed overall high classification, average accuracy, and kappa coefficients. The reason behind this was that the proposed MFO method selected the best-suitable bands compared to other optimization methods and the selected bands had low entropy. Figure 9a shows the convergence curve of the Indian Pines dataset. Here, the MFO-based band selection had after the 130th iteration an overall accuracy of 88.98%. For the University of Pavia dataset, the optimal global solution was attained at the 126th iteration with an accuracy of 93.92% and is shown in Figure 9b. At the 132nd iteration, the Salinas dataset attained its optimum global solution with an accuracy of 96.94% and is shown in Figure 9c. This implies that MFO is the best appropriate solution for the selection of hyperspectral bands.

### 5.2. Computation Time

The computational complexity of the MFO method relies on several factors such as the number of moths, the number of variables, the maximum number of iterations, and the sorting technique of flames in each iteration [34]. Since we employed the Quicksort method, $O\left(nlogn\right)and\;O\left(n^{2}\right)$ in the best and worst case, correspondingly. Considering the P function, consequently, the entire computational complexity is determined by,

(9) $$O\left(MFO\right)=O\left(t\left(n^{2}+n\right)\right)=O\left(tn^{2}+tnd\right)$$

where *n* is the number of moths, *t* is the maximum number of iterations, and d is the number of variables. In this paper, the maximum value of the combined pixels was considered as n and 200 iterations, and the number of variables represents the number of bands present in the hyperspectral data. Table 9 summarizes the processing time needed by each procedure on each of the three datasets. The overall execution time is the amount of time taken to complete the band collection and classification process. As the table indicates, the suggested approach took a shorter time than the other methods to compute the classifier’s overall accuracy. While the proposed approach required more time to execute, it achieved a high degree of classification accuracy. Table 8 describes the overall comparison of our proposed work with PSO, GAO, and cuckoo search. The results show that the MFO algorithm achieved higher accuracies with the minimum iterations to obtain the global minimum.

Mirjalili [34] mentioned that the MFO algorithm can theoretically be more effective in solving optimization problems, so we observed that this algorithm improved the performance of the HSI band selection method compared with all the other methods. MFO processes the updating positions, allowing acquiring neighboring bands surrounding the flames, a mechanism for largely encouraging exploitations. Local optimum avoidance was high because MFO uses a population of moths. There is no way for the greatest bands to be lost because the F matrix stores them. Exploration and exploitation are balanced using an appropriate number of flames.

## 6. Conclusions

In this paper, band selection was performed by the optimization of an objective function. The classification accuracy rate and the class separability measure were combined in the design procedure of the respective objective function. A two-class separability measure, which was the K-means distances, was proposed in this method. A new meta-heuristic called the moth–flame optimizer was used to optimize the objective function, providing better results. The proposed approach was tested on three widely used HSI datasets: University of Pavia, Indian Pines, and Salinas Scene, to show the effectiveness. Effectiveness was measured in terms of overall accuracy, average accuracy, individual class accuracy, and computational time. A comparison with some feature selection methods that are defined in the literature was also conducted. The obtained classification accuracy rate of the proposed approach was very satisfactory compared to the others. In this approach, 80% of the data were used to train the algorithm. The proposed technique selects the better band that separates the classes, thereby increasing the classification rate. In the future, the same proposed algorithm can be tested for different hyperspectral datasets, and the quality of the objective function can also be worked on for improvement.

More extensive comparisons incorporating class accuracies and other statistics, as well as tests with more HSI datasets are required to fully appreciate MFO’s benefits. Future research will look at other datasets and compare MFO to other cutting-edge metaheuristic algorithms to further understand its efficacy. Experimenting with newer heuristics such as MFO for HSI band selection is vital since the no free lunch theorem asserts that different optimization methods will be better for different situations and that there is no one optimal strategy for all challenges. 

## Figures and Tables

**Table 1:** Band selection using different optimization techniques.

Optimization Techniques	Airborne Hyperspectral Dataset	Non-Optimized Bands	Number of Non-Optimized Band
**MFO**	Indian Pines	[0, 1, 2, 4, 5, 6, 9, 57, 76, 79, 101, 102, 104, 141, 143, 191]	16
	Salinas	[0, 1, 2, 3, 4, 5, 103, 104, 105, 106, 108, 146, 147]	13
	Pavia University	[0, 1, 2, 3, 6, 12, 15, 24, 26, 85, 94]	11
**PSO**	Indian Pines	[0, 1, 2, 3, 4, 5, 6, 9, 57, 76, 79, 101, 102, 103, 104, 143, 144, 145, 191]	19
	Salinas	[0, 1, 2, 3, 4, 16, 60, 79, 80, 105, 146, 155, 152]	13
	Pavia University	[0, 1, 3, 4, 5, 6, 11, 12, 24, 27, 44, 85, 94, 98, 101]	15
**GAO**	Indian Pines	[0, 1, 2, 4, 5, 6, 7, 9, 16, 33, 41, 47, 57, 75, 82, 101, 104, 113, 124, 139, 144, 186, 181, 183]	24
	Salinas	[0, 1, 2, 3, 4, 5, 6, 8, 31, 56, 102, 103, 106, 108, 144, 145, 157, 159, 161]	19
	Pavia University	[0, 1, 2, 3, 4, 5, 8, 12, 14, 15, 22, 26, 28, 36, 68, 86, 91, 101]	18
**CSO**	Indian Pines	[0, 1, 4, 5, 7, 19, 41, 76, 77, 84, 89, 95, 100, 101, 102, 104, 143, 144, 174, 175, 184, 192]	22
	Salinas	[0, 1, 2, 3, 4, 5, 8, 9, 21, 34, 46, 82, 86, 103, 104, 105, 107, 145, 146, 147]	20
	Pavia University	[0, 1, 2, 3, 4, 5, 6, 11, 14, 24, 26, 56, 57, 71, 84, 86, 94]	17

**Table 2:** Details of the datasets—Indian pines, University of Pavia, and Salinas datasets used in this study.

Indian Pines Hyperspectral Data				University of Pavia Hyperspectral Data				Salinas Scene Hyperspectral Data			
**Class Name**	**Training** **Samples**	**Test** **Samples**	**Total** **Samples**	**Class Name**	**Training** **Samples**	**Test** **Samples**	**Total** **Samples**	**Class Name**	**Training** **Samples**	**Test** **Samples**	**Total** **Samples**
Alfalfa	9	36	45	Asphalt	1314	5256	6570	Broccoli green weeds 1	419	1676	2095
Corn-notill	283	1130	1413	Meadows	3725	14,898.4	18,623	Broccoli green weeds 2	737	2948	3685
Corn-mintill	169	674.4	843	Bitumen	420	1680	2100	Fallow	390	1558	1948
Corn	45	180	225	Gravel	619	2476	3095	Fallow rough plow	285	1140	1425
Grass-pasture	95	378.4	473	Bare Soil	286	1144	1430	Fallow smooth	559	2236	2795
Grasstrees	143	570.4	713	Painted metal sheets	1008	4030.4	5038	Stubble	817	3268	4085
Grass- pasture- mowed	6	24	30	Self-Blocking Bricks	269	1076	1345	Celery	703	2810	3513
Hay- windrowed	91	364	455	Shadows	729	2916	3645	Grapes untrained	2205	8820	11025
Oats	3	10.4	13	Trees	187	746.4	933	Soil vineyard develop	1231	4924	6155
Soybean-notill	206	822.4	1028					Corn senesced green weeds	659	2634	3293
Soybean-mintill	484	1936	2420					Lettuce romaine 4 wk	199	794	993
Soybean-clean	123	490.4	613					Lettuce romaine 5 wk	383	1530	1913
Wheat	41	164	205					Lettuce romaine 6 wk	176	702	878
Woods	259	1036	1295					Lettuce romaine 7 wk	207	826	1033
Buildings Grass Trees Drives	78	312	390					Vineyard untrained	1482	5928	7410
Stone Steel Towers	19	74.4	93					Vineyard vertical trellis	378	1510	1888

**Table 3:** Comparison of classwise accuracies (in%) of MFO with the state-of-the-art methods on Indian Pines using RF, KNN, and SVM.

Class Names of Indian Pines Dataset	RF				KNN				SVM			
	PSO	GAO	CSO	MFO	PSO	GAO	CSO	MFO	PSO	GAO	CS	MFO
Alfalfa	61.11	55.56	66.67	61.11	55.56	55.56	61.11	72.22	94.44	94.44	88.89	**100**
Corn-notill	76.81	77.88	78.05	76.46	70.09	68.85	70.80	69.91	80.35	80.71	**81.77**	80.18
Corn-mintill	63.20	56.97	61.42	63.20	60.53	58.46	59.94	60.53	81.01	81.90	**82.20**	81.31
Corn	37.78	33.33	34.44	36.67	37.78	41.11	50	37.78	82.22	86.67	81.11	**82.22**
Grass-pasture	88.36	95.24	93.12	91.53	92.06	92.59	92.59	92.06	96.30	96.83	96.83	**96.85**
Grasstrees	97.89	98.25	97.54	97.54	97.19	96.84	97.54	97.19	97.89	97.54	97.82	**100**
Grass-pasture-mowed	58.33	75	50	66.67	75	66.67	75	100	83.33	83.33	83.33	**100**
Hay-windrowed	99.45	100	99.45	100	98.35	97.80	98.90	100.55	100	99.45	99.45	**100**
Oats	100	60	40	60	80	60	80	100	100	100	100	**100**
Soybean-notill	76.89	76.64	77.86	78.35	80.78	80.78	82.24	80.78	**85.16**	84.43	81.75	84.18
Soybean-mintill	90.81	89.88	90.19	**90.70**	79.96	79.75	79.75	80.06	89.67	90.08	90.08	89.77
Soybean-clean	75.92	75.10	74.29	74.29	48.98	48.98	49.80	48.98	87.76	86.12	85.71	**88.16**
Wheat	97.56	97.56	97.56	98.78	95.12	93.90	96.34	95.12	98.78	97.56	98.78	**100**
Woods	**98.26**	97.30	96.91	97.68	93.44	94.21	93.44	93.44	96.72	97.10	97.10	96.72
Buildings Grass Trees Drives	62.18	58.97	61.54	58.33	39.10	34.62	38.46	39.10	**78.21**	73.72	76.92	**78.21**
Stone Steel Towers	89.19	89.19	89.19	89.19	**91.89**	89.19	91.89	91.89	86.49	**91.89**	86.49	86.49

**Table 4:** Comparison of classwise accuracies (in %) of MFO with state-of-the-art methods on the University of Pavia dataset using RF, KNN, and SVM.

Class Name of University of Pavia Data	RF				KNN				SVM			
	PSO	GAO	CSO	MFO	PSO	GAO	CSO	MFO	PSO	GAO	CS	MFO
Asphalt	95.81	96.84	95.81	96.12	91.48	91.36	91.40	95.81	95.81	95.74	91.48	**96.69**
Meadows	98.13	98.34	98.44	98.46	98.35	98.43	98.32	98.43	98.43	98.50	98.35	**98.67**
Bitumen	78.45	73.33	78.69	71.19	75.83	77.26	75.60	78.45	78.45	78.81	75.83	76.43
Gravel	96.93	94.10	**97.25**	94.18	89.10	89.34	89.26	96.93	96.93	97.50	89.10	94.10
Bare Soil	100	99.83	**100**	**100**	99.65	99.65	99.48	**100**	100	100	99.65	100
Painted metal sheets	92.31	88.68	91.36	86.55	76.77	77.57	76.43	92.31	92.31	91.56	76.77	86.90
Self-Blocking Bricks	86.80	76.21	86.62	77.70	88.66	88.10	87.36	86.80	86.80	86.43	88.66	86.80
Shadows	93.07	93.14	92.87	92.59	88.82	90.19	88.89	93.07	93.07	93.07	88.82	93.21
Trees	100	100	100	100	100	100	100	100	100	100	100	100

**Table 5:** Comparison of classwise accuracies (in %) of MFO with the state-of-the-art methods on Salinas Scene dataset using RF, KNN, and SVM.

Class Name of Salinas Scene Data	RF				KNN				SVM			
	PSO	GAO	CSO	MFO	PSO	GAO	CSO	MFO	PSO	GAO	CSO	MFO
Broccoli green weeds 1	99.16	99.18	99.18	99.18	98.69	98.69	98.69	98.69	99.14	98.01	98.17	**99.52**
Broccoli green weeds 2	**100**	99.93	99.93	**100**	99.73	99.73	99.73	99.73	**100**	**100**	**100**	**100**
Fallow	**100**	99.87	**100**	99.87	100	**100**	**100**	**100**	**100**	**100**	**100**	**100**
Fallow rough plow	99.82	99.82	99.82	99.82	99.82	99.82	99.82	99.82	**100**	**100**	**100**	**100**
Fallow smooth	99.73	99.55	**99.73**	99.55	98.57	98.57	98.48	98.48	99.46	99.55	99.46	99.55
Stubble	**99.94**	98.86	98.17	98.41	99.76	99.76	99.76	99.76	98.12	99.87	99.13	**99.94**
Celery	98.32	99.71	99.64	99.72	99.36	99.29	99.36	99.36	**99.72**	**99.72**	**99.72**	**99.72**
Grapes untrained	**93.40**	**93.58**	**93.56**	**93.58**	85.78	85.60	85.56	85.78	90.77	90.86	90.77	91.02
Soil vineyard develop	99.96	**100**	99.96	99.96	99.59	99.63	99.63	99.63	99.92	99.92	99.92	99.92
Corn senesced green weeds	98.48	98.86	98.25	98.24	94.38	94.31	94.46	94.31	98.71	98.48	**98.63**	**98.63**
Lettuce romaine 4 wk	98.49	**99.50**	98.74	98.99	97.98	97.98	97.98	97.98	99.24	98.49	98.74	98.49
Lettuce romaine 5 wk	**100**	**100**	**100**	**100**	99.87	99.87	**100**	99.87	**100**	**100**	**100**	**100**
Lettuce romaine 6 wk	98.01	98.01	98.01	98.58	97.72	97.72	97.72	97.72	99.43	99.43	99.43	**99.43**
Lettuce romaine 7 wk	98.55	98.31	98.31	97.82	95.40	95.40	95.40	95.40	98.14	97.69	96.13	**98.79**
Vineyard untrained	**78.58**	78.37	77.63	77.67	69.80	68.93	69.47	69.43	71.29	70.72	70.38	70.82
Vineyard vertical trellis	98.94	98.94	98.94	98.94	98.54	98.41	98.41	98.41	**99.07**	98.94	**99.07**	98.94

**Table 6:** Overall and average accuracy measures and Kappa coefficient measure for all the classifiers with comparative analysis of state-of-the-art approaches for Indian Pines data.

Class Name of Indian Pines Data	RF				KNN				SVM			
	PSO	GA	CS	MFO	PSO	GA	CS	MFO	PSO	GA	CS	MFO
Overall Accuracy	83.68	83.02	83.41	83.54	77.32	76.80	77.88	77.51	88.90	88.93	88.78	88.98
Average Accuracy	79.61	77.30	75.51	77.18	74.74	72.46	76.11	77.68	89.90	90.11	89.27	91.37
Kappa Coefficient	0.81	0.81	0.81	0.81	0.74	0.74	0.75	0.75	0.87	0.88	0.87	0.88

**Table 7:** Overall and average accuracy measures and Kappa coefficient measure for all the classifiers with comparative analysis of state-of-the-art approaches for University of Pavia data.

Class Name of University of Pavia Data	RF				KNN				SVM			
	PSO	GA	CS	MFO	PSO	GA	CS	MFO	PSO	GA	CS	MFO
Overall Accuracy	93.98	94.38	93.99	93.98	91.94	92.24	91.84	94.62	95.48	95.46	95.39	96.94
Average Accuracy	90.75	91.16	90.64	90.75	89.85	90.21	89.64	92.49	93.53	93.51	93.45	94.85
Kappa Coefficient	0.92	0.93	0.92	0.92	0.89	0.90	0.89	0.93	0.94	0.94	0.94	0.89

**Table 8:** Overall and average accuracy measures and Kappa coefficient measure for all the classifiers with comparative analysis of state-of-the-art approaches for Salinas Scene data.

Class Name of Salinas Scene Data	RF				KNN				SVM			
	PSO	GA	CS	MFO	PSO	GA	CS	MFO	PSO	GA	CS	MFO
Overall Accuracy	95.45	95.49	95.34	95.39	92.16	91.99	92.07	92.10	93.95	93.82	93.87	93.92
Average Accuracy	97.71	97.77	97.65	97.70	95.94	95.86	95.90	95.90	97.24	97.15	97.15	97.17
Kappa Coefficient	0.95	0.95	0.95	0.95	0.91	0.91	0.91	0.91	0.93	0.93	0.93	0.93

**Table 9:** Runtime (in seconds) needed for the datasets and methods.

Optimization Techniques		Random Forest	K-Nearest Neighbor	Support Vector Machine
Indian Pines	MFO	165	184	206
	CSO	188	172	195
	GAO	348	647	647
	PSO	557	628	722
University of Pavia	MFO	714	768	802
	CSO	644	704	785
	GAO	984	1084	1102
	PSO	1023	1069	1248
Salinas Scene	MFO	897	831	1046
	CSO	974	1003	1086
	GAO	1142	1340	1424
	PSO	1267	2018	2123

## Data Availability

Not applicable.
